# Supplementary material for: Lactiplantibacillus plantarum 1008 Enhances Testicular Function and Spermatogenesis via the Modulation of Gut Microbiota in Male Mice with High-Fat-Diet-Induced Obesity
Source: Biology (Basel). 2024 Oct 31;13(11):890. doi: 10.3390/biology13110890 (PMC11592197; doi:10.3390/biology13110890)
Supplement: Supplementary file 1 [file biology-13-00890-s001.zip › biology-3252161-supplementary.pdf]

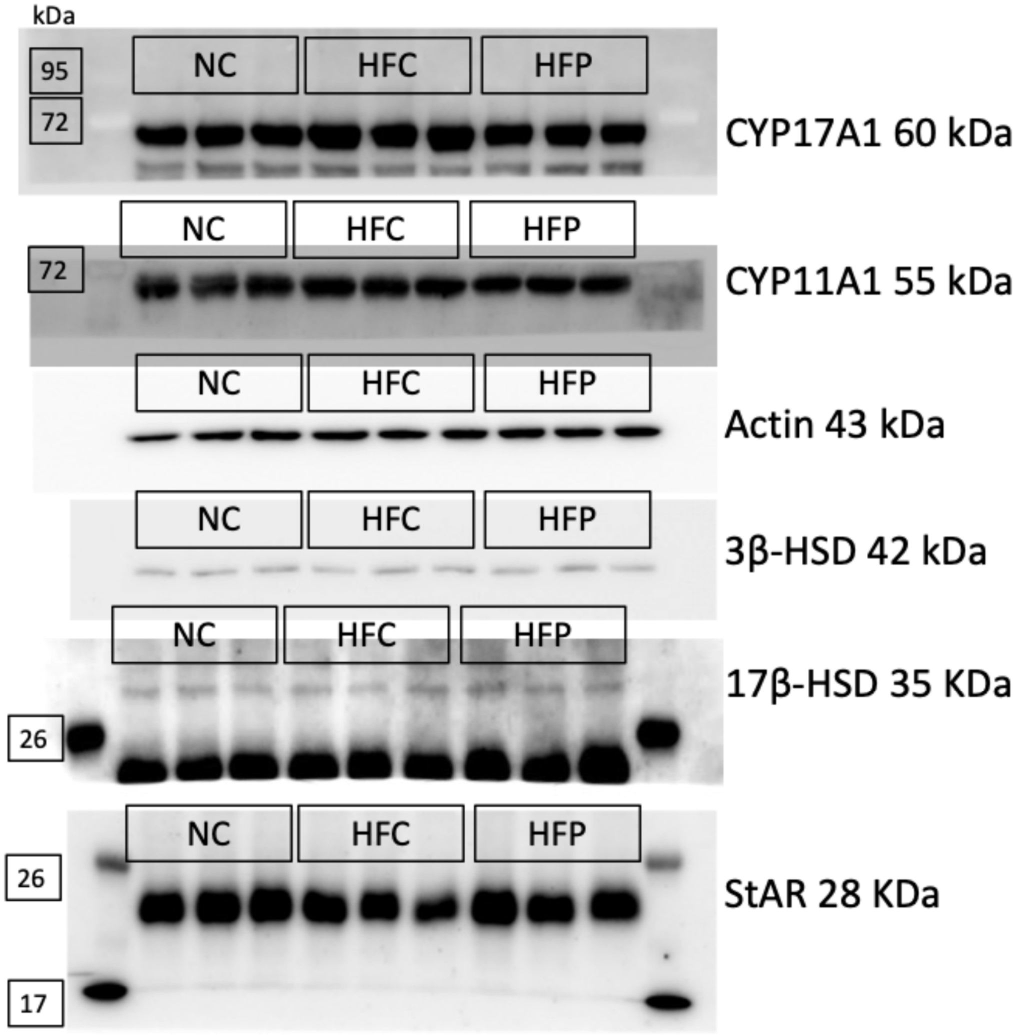

Figure S1. Whole western blot of CYP17A1 (~60 kDa), CYP11A1 (~55 kDa), Actin (~43 kDa), 3β-HSD (~42 kDa), 17β-HSD (~35 kDa) and StAR (~28 kDa) detected with antibodies after cutting membrane in high-fat diet and *LP1008* treatment in male mice.

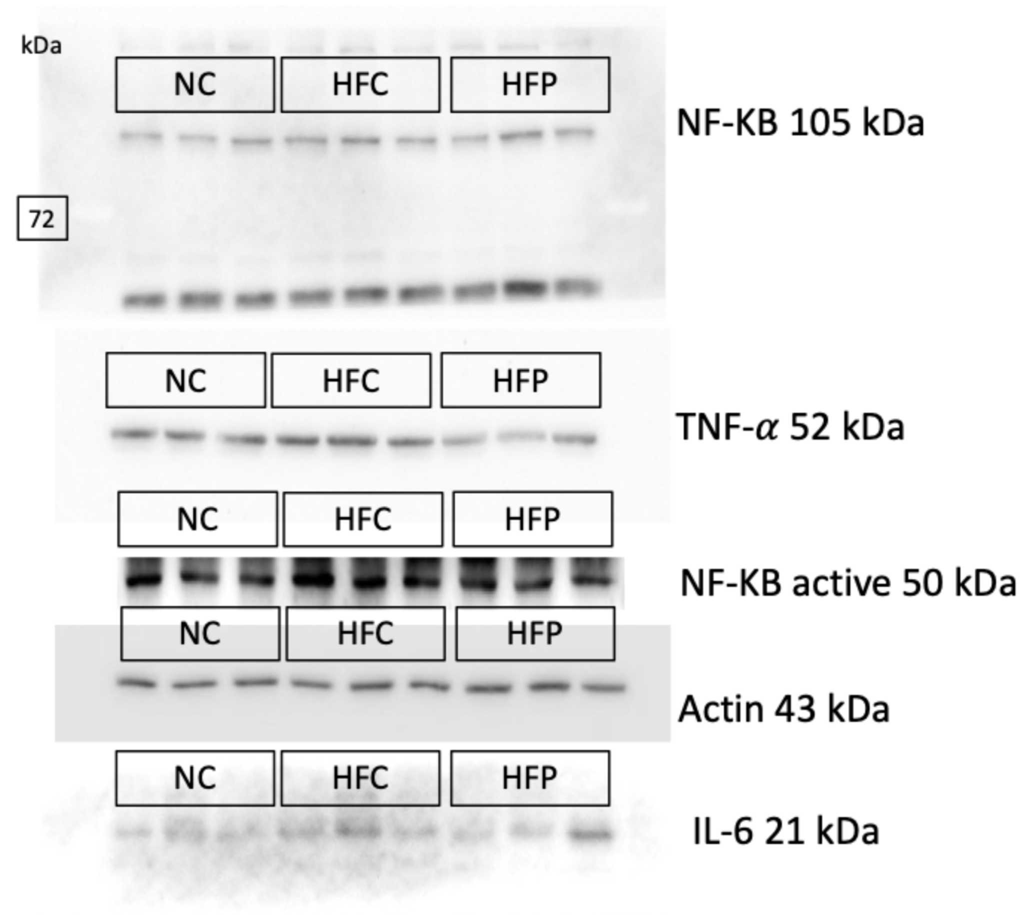

Figure S2. Whole western blot of NF-KB (105 kDa), TNF-α (52 kDa), NF-KB active (50 kDa), Actin (43 kDa) and IL-6 (21 kDa) detected with antibodies after cutting membrane in high-fat diet and *LP1008* treatment in male mice.

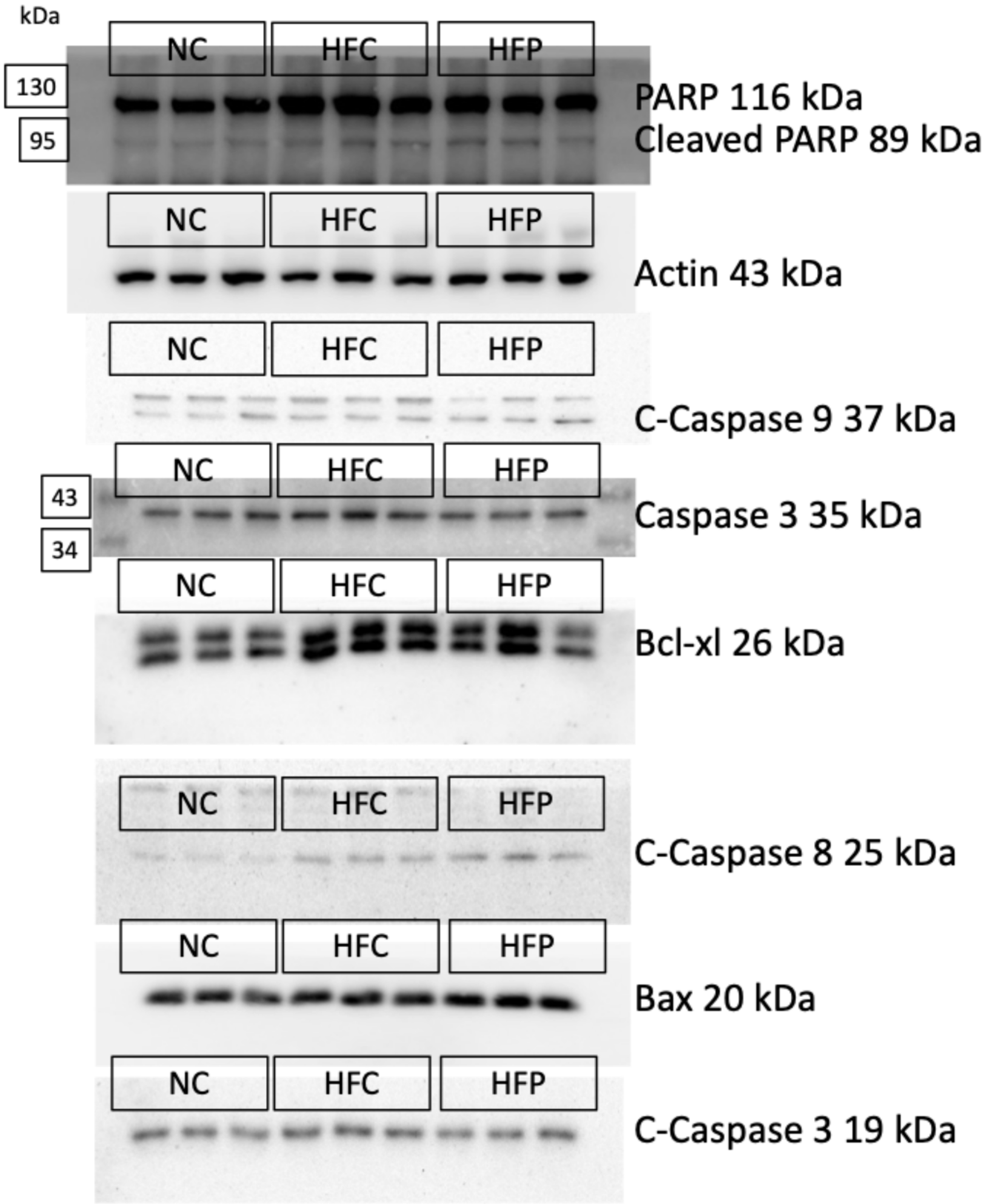

Figure S3. Whole western blot of PARP (116 kDa), Cleaved PARP (89 kDa), Actin (43 kDa), C-Caspase 9 (37 kDa), Caspase 3 (35 kDa), Bcl-xl (26 kDa), C-Caspase 8 (25 kDa), Bax (20 kDa) and C-Caspase 3 (19 kDa) detected with antibodies after cutting membrane in high-fat diet and *LP1008* treatment in male mice.

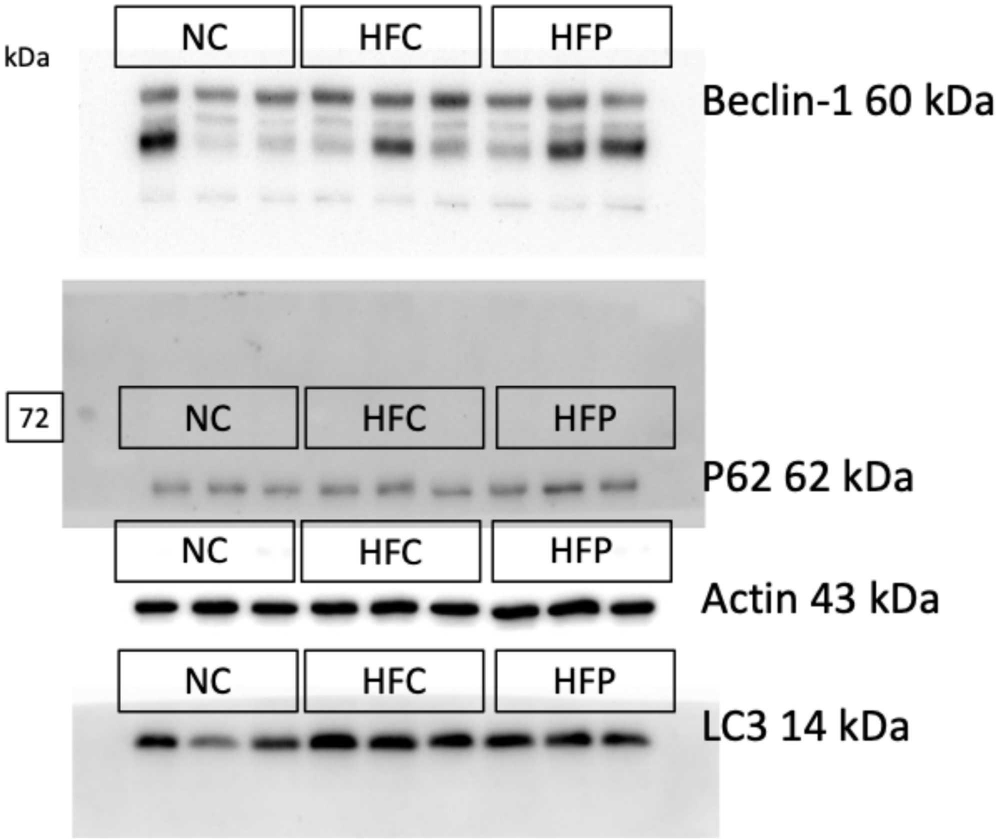

Figure S4. Whole western blot of Beclin-1 (60 kDa), P62 (62 kDa), Actin (43 kDa) and LC3 (14 kDa) detected with antibodies after cutting membrane in high-fat diet and *LP1008* treatment in male mice.
